# Expert validation of fit-for-purpose guidelines for designing programmes of assessment

**DOI:** 10.1186/1472-6920-12-20

**Published:** 2012-04-17

**Authors:** Joost Dijkstra, Robert Galbraith, Brian D Hodges, Pauline A McAvoy, Peter McCrorie, Lesley J Southgate, Cees PM Van der Vleuten, Val Wass, Lambert WT Schuwirth

**Affiliations:** 1 Department of Educational Development and Research, Maastricht University, Maastricht, The Netherlands; 2 Center for Innovation, National Board of Medical Examiners, Philadelphia, USA; 3 Wilson Centre for Research in Education, Faculty of Medicine, University of Toronto, Toronto, ON, Canada; 4 Assessment Development, National Clinical Assessment Service (NCAS), London, UK; 5 Centre for Medical and Healthcare Education, St George’s, University of London, London, UK; 6 Keele University, School of Medicine, Staffordshire, UK; 7 Flinders Innovation in Clinical Education, Flinders University, Bedford Park, SA, Australia

## Abstract

**Background:**

An assessment programme, a purposeful mix of assessment activities, is necessary to achieve a complete picture of assessee competence. High quality assessment programmes exist, however, design requirements for such programmes are still unclear. We developed guidelines for design based on an earlier developed framework which identified areas to be covered. A fitness-for-purpose approach defining quality was adopted to develop and validate guidelines.

**Methods:**

First, in a brainstorm, ideas were generated, followed by structured interviews with 9 international assessment experts. Then, guidelines were fine-tuned through analysis of the interviews. Finally, validation was based on expert consensus via member checking.

**Results:**

In total 72 guidelines were developed and in this paper the most salient guidelines are discussed. The guidelines are related and grouped per layer of the framework. Some guidelines were so generic that these are applicable in any design consideration. These are: the principle of proportionality, rationales should underpin each decisions, and requirement of expertise. Logically, many guidelines focus on practical aspects of assessment. Some guidelines were found to be clear and concrete, others were less straightforward and were phrased more as issues for contemplation.

**Conclusions:**

The set of guidelines is comprehensive and not bound to a specific context or educational approach. From the fitness-for-purpose principle, guidelines are eclectic, requiring expertise judgement to use them appropriately in different contexts. Further validation studies to test practicality are required.

## Background

There is a growing shared vision that a *programme* of assessment is necessary to achieve a coherent and consistent picture of (assessee) competence [1-4]. A programme is more than a combination of separate tests. Just as a test is not simply a random sample of items; a programme of assessment is more than a random set of instruments. An optimal mix of instruments should match the purpose of assessment in the best possible way. However, there is less clarity about what is actually needed to achieve an integrated, high quality programme of assessment. Little is known about key relations, compromises, and trade-offs needed at the level of a highly integrated programme of assessment [5]. This does not imply that existing programmes of assessment are not of high quality, indeed there are numerous examples of good programmes of assessment which are based on extensive deliberation and which are designed by experts [6-8].

However, scientific evidence on quality of such programmes in its entirety is currently limited, and certainly in need of theory formation and applicable research outcomes. The scant research that has been conducted into the quality of programmes of assessment, focuses on various aspects of assessment, with different aims and adopting diverse viewpoints on quality, and the results of the individual studies therefore are hard to compare. From a psychometric perspective quality has been almost exclusively defined as the reliability of combinations of decisions and a “unified view of validity” [9-13]. From an educational perspective the focus has been on the alignment of objectives, instruction, and on using assessment to stimulate desirable learning behaviour [14-16]. In another study Baartman [17] took competency-based education as a basis for quality, and proposed adding education-based criteria, such as authenticity and meaningfulness, to the established psychometric criteria. Most of this research determines assessment quality afterwards, when assessment has already taken place. Unfortunately, this does not provide assessment designers with much support when they intend to construct a high-quality programme. In our study we therefore investigate the possibility of enhancing quality of assessment programmes from a design perspective by providing guidelines for assessment design.

In various local contexts standards, criteria, and guidelines are used to support assessment development. However, the transferability of these to other contexts is fairly low as they are highly contextual and often based on local policy decisions. On the other hand guidance is available at a broader educational level, e.g., the Standards for educational and psychological testing [18]. But these standards focus predominantly on single tests (i.e. the measuring instrument) instead of on programmes of assessment. And, despite the standards being open to expert judgement and acknowledging contextual differences (e.g. in regulations), they are still formulated from a specific testing framework and from the perspective of *assessment of learning*[19]. This predetermines the goal of assessment and takes an ideological standpoint in the quality perspective and as a result, such standards are necessarily prescriptive. So, our aim in this study is to develop and validate more context-independent guidelines, applicable with different purposes in mind (including *assessment for learning*), and with a focus on programmes of assessment instead of single instruments. In addition we seek to develop and validate guidelines that support both assessment developers and decision makers. In this study we adopted the *fitness-for-purpose* principle [5,20], in which quality is determined as the extent to which a programme of assessment fulfils its purpose or its function. The advantage of this is that it makes the quality framework more widely applicable and less reliant on contemporary ideas on education and assessment. From the fitness-for-purpose perspective defining *criteria* is avoided, and instead *design guidelines* are formulated. For example, a quality criterion would be: “An assessment programme should have summative tests”, whereas a guideline would be: “The need for summative tests should be considered in light of the purpose.” Given the fitness-for-purpose principle the application of the guidelines are necessarily eclectic. In different contexts assessment designers need to decide how important or relevant a guideline is, and use their own expertise to make decisions based on specific contextual circumstances.

**Figure 1:**
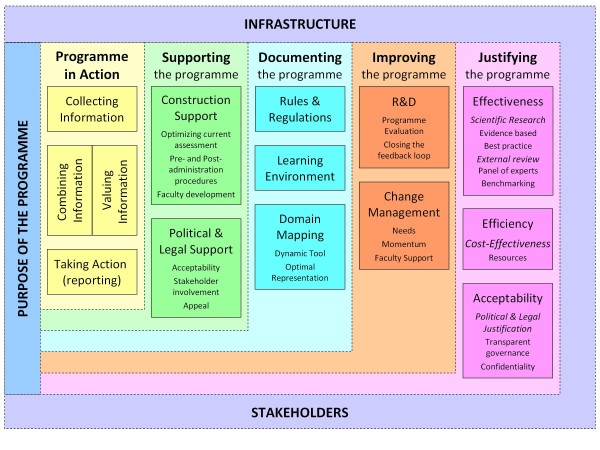
Framework for programmes of assessment.

In this paper we propose a set of design guidelines for programmes of assessment, based on a framework developed in our previous research [5]. This framework defines the scope of what constitutes a programme of assessment and should be covered by our guidelines (see Figure 1).

The framework is divided into several layers and is placed in the context of *stakeholders* and *infrastructure* (outer layer). The starting point is the *purpose of the programme* (key element in the framework). Around the purpose, 5 layers (dimensions) were distinguished. (1) *Programme in action* describes the core activities of a programme, i.e. collecting information, combining and valuing the information, and taking subsequent action. (2) *Supporting the programme* describes activities that are aimed at optimizing the current programme of assessment, such as improving test construction and faculty development, as well as gaining stakeholder acceptability and possibilities for appeal. (3) *Documenting the programme* describes the activities necessary to achieve a defensible programme and to capture organizational learning. Elements of this are: rules and regulations, learning environment, and domain mapping. (4) *Improving the programme* includes dimensions aimed at the re-design of the programme of assessment, after the programme is administered. Activities are R&D and change management. (5) The final layer *justifying the programme* describes activities that are aimed at providing evidence that the purpose of the programme is achieved taking account of effectiveness, efficiency, and acceptability.

Because the aim of this study was to formulate guidelines that are general enough to be applicable to a variety of contexts, and yet at the same time meaningful and concrete enough to support assessment designers, we started by generating ideas for guidelines based on the above framework for programmes of assessment using the input of international experts in the field of assessment in medical education. In order to validate the guidelines we sought expert consensus. In this article we do not go into further detail about the framework; but kindly refer the reader to our previous publication [5]. In describing the results we will focus on the most important and salient findings (i.e. the guidelines). For the complete set of guidelines we refer to Additional file 1: the addendum.

## Method

### Study design

The development and validation of design guidelines was divided into four phases, starting with a brainstorm phase to generate ideas using a core group of experts (JD, CvdV and LWTS), followed by a series of discussions with a wider group of international experts to elaborate on this brainstorm. Next in a refinement phase, the design guidelines were fine-tuned based on the analysis of the discussions. Finally a member check phase was initiated to validate the guidelines based on expert consensus.

### Participants

The participants were purposefully selected based on their experience with programmes of assessment. They all have published extensively on assessment. Given their backgrounds it was anticipated that these experts would provide the most valuable information. The nine participants of the focus group of the preceding study [5] were invited by e-mail to participate in this follow-up study, explaining the goal and providing details about the method and procedures. One participant declined because of retirement, another declined because of other obligations, a third declined because of a change in field of work. With the addition of CvdV and LWTS a total of eight experts took part in this study. The experts (all co-authors) came from North America (2) and Europe (6). Within their institution, they fulfil different (and some multiple) roles in their assessment practice e.g. programme directors, national committee members, and other managerial roles. They represent different (educational) domains ranging from undergraduate and graduate education, to national licensing and recertification.

### Procedure and data analysis

The brainstorm was done by the research team (JD, CvdV, LWTS) based on their experience and data from the preceding study [5]. This resulted in a first draft of the set of guidelines, which served as a starting point for the discussion phase. The discussion took place in multiple (Skype®) interviews with the participants. Individual interviews were held with each participant and led by one researcher (JD) with the support of a second member of the research team (either CvdV or LWTS). The interview addressed the first draft of guidelines and was structured around three open questions: 1. Is the formulation of the guidelines clear, concise, correct? 2. Do you agree with the guidelines? 3. Are any specific guidelines missing? The interviews were recorded and analysed by the research team to distil a consensus from the various opinions, suggestion, and recommendations. One researcher (JD) reformulated the guidelines and to avoid overly adherence to initial formulations the interview data (expert suggestions) were taken as starting point. The goal of the new formulation was to represent the opinions and ideas expressed by the experts as accurately as possible. Peer debriefing was done to check the reformulation by the research team (JD, CvdV, & LWTS) to reach initial consensus. After formulating a complete and comprehensive set of guidelines, a member-check procedure was conducted by e-mail. All participants were sent the complete set for final review and all responded. No content-related issues had to be resolved and some wording issues were resolved as a final consensus document was generated.

## Results

A set of 72 guidelines was developed based on expert experience, and then validated based on expert consensus. Because of the length of this list we have decided not to provide exhaustive detail about all of them, but to limit ourselves to the most salient guidelines per layer of the framework (the complete list is provided as an addendum in Additional file 1). For reasons of clarity, a few remarks on how to read this section and the addendum with the complete set of guidelines. Firstly, the guidelines are divided over the layers of the framework and grouped per element within each layer. We advise the reader to regard the guidelines in groups rather than as separate guidelines. Also in application of the guidelines it is expected that it is not practical to apply guidelines in isolation. Secondly, there is no linear order in the guidelines presented. When reading the guidelines, you may not immediately come across those guidelines or important topics you would expect to be given priority. There is potentially more than one way of ordering the guidelines. For instance *costs* are important throughout the design process. However, because of the way this framework is constructed, *costs* are addressed near to the end. Thirdly, there is overlap in the guidelines. It appeared impractical and somewhat artificial to split every assessment activity into separate parts. The guidelines are highly related, and overlap and/or redundancy are almost inevitable. In the example of *costs*, which are primarily addressed as part of *cost-efficiency*, references to *costs* are actually made in several guidelines. Fourthly, the level of granularity is not equal for all guidelines. Determining the right level of detail is a difficult endeavour, variable granularity reflects the fact that some issues seem more important than others, and others may have been investigated in depth. Hence, the interrelatedness and the difficulty of determining the right level of granularity is also a reason to review the guidelines per group. The division of guidelines within elements of the layers was done based on key recommendations in the design process. However, in some situations this division might be arbitrary and of less relevance. Finally we have sought to find an overarching term that would cover all possible elements of the programme, such as assessments, tests, examinations, feedback, and dossiers. We wanted the guidelines to be broadly applicable, and so we have chosen the term assessment components. Similarly for outcomes of assessment components we have chosen assessment information (e.g. data about the assessees’ competence or ability).

### General

In addition to the fact that the number of guidelines exceeded our initial expectations, we found that most guidelines focused on the more practical dimensions of the framework (see Table 1). In particular, many of the guidelines deal with *collecting information*. This is not unexpected, since considerable research efforts are focused on specific assessment components for collecting information (measuring). On the other hand some guidelines (e.g. on *combining information*) are less explicit and straightforward and there is less consensus, resulting in less nuanced guidelines.

**Table 1:** Number of guidelines per layer

**Layer**	**Number of guidelines**
**Purpose** **Infrastructure** **Stakeholder**	**3** **2** **2**
**Programme in Action** ** ➣Collecting information** ** ➣Combining information** ** ➣Valuing information** ** ➣Taking Action**	**21** ** ➣13** ** ➣3** ** ➣2** ** ➣3**
**Supporting the Programme** ** ➣Construction Support** ** ➣Political Support**	**12** ** ➣5** ** ➣7**
**Documenting the Programme** ** ➣Rules and Regulations (R&R)** ** ➣Learning Environment** ** ➣Domain Mapping**	**12** ** ➣6** ** ➣2** ** ➣4**
**Improving the programme** ** ➣R&D** ** ➣Change Management**	**7** ** ➣3** ** ➣4**
**Justifying the Programme** ** ➣Scientific research** ** ➣External Review** ** ➣Efficiency** ** ➣Acceptability**	**10** ** ➣2** ** ➣2** ** ➣2** ** ➣4**

Three major principles emerged and led to generic guidelines that are applicable in any design consideration are set out below. These are (1) the principle of proportionality, (2) the need to substantiate decisions applying the fitness-for-purpose principle, and (3) getting the right person for the right job. These were translated into the following general guidelines (I-III):

I). *Decisions (and their consequences) should be proportionate to the quality of the information on which they are based.*

This guideline has implications for all aspects of the assessment programme, both at the level of the design of the programme, and at the level of individual decisions about assessees’ progress. The higher the stakes, the more robust the information needs to be.

In the layer *Programme in Action* for instance, actions based on (collected) information should be proportionate to the quantity and quality of the information. The more high-stakes an action or decision, the more certainty (justification and accountability) is required, the more the information collection process has to comply with scientific criteria, and usually the more information that is required.

For example the decision that an assessee has to retake one exam, can be taken based on less information (e.g. the results of one single test) compared to a decision that the assessee has to retake an entire year of medical school, which clearly requires a series of assessments or maybe even a dossier.

II) *Every decision in the design process should be underpinned preferably supported by scientific evidence or evidence of best practice. If evidence is unavailable to support the choices made when designing the programme of assessment, the decisions should be identified as high priority for research.*

This implies that all choices made in the design process should be defensible and can be justified. Even if there is no available scientific evidence, a plausible or reasonable rationale should be proposed. Evidence can be sought through a survey of the existing literature, new research endeavours, collaborative research, or completely external research. We stress again that the fitness-for-purpose principle should guide design decisions. The evaluation of the contribution to achieving the purpose(s) should be part of the underpinning.

III) *Specific expertise should be available (or sought) to perform the activities in the programme of assessment.*

This guideline is more specifically aimed at the expertise needed for the assessment activities in the separate layers and elements within the assessment programme. A challenge in setting up a programme of assessment is to “get the right person for the right job”. Expertise is often needed from different fields including specific domain knowledge, assessment expertise, and practical knowledge about the organisation. Some types of expertise, such as psychometric expertise for item analysis, and legal expertise for rules and regulations, are obvious. Others are less clear and more context specific. It is useful when designing an assessment programme to articulate the skill set and the body of knowledge necessary to address these issues.

### Salient guidelines per dimensions in the framework

This section contains the more detailed and specific guidelines. We describe them in relation to the layers of our previously described model (see Figure 1), starting from the *purpose* towards the outer layers. In the addendum ( Additional file 1) all guidelines are described and grouped per element within each layer.

### Purpose, stakeholders, and infrastructure

From the fitness for purpose perspective, by definition the purpose of an assessment programme is an important key element. The authors all agreed that defining the purpose of the programme of assessment is essential and must be addressed at a very early stage of the (re)design. Although there was some initial debate on the level of detail and the number of purposes, it was generally acknowledged that, at least in theory, there should be one principal purpose.

A1 *One principal purpose of the assessment programme should be formulated.*

This principal purpose should contain the function of the assessment programme and the domains to be assessed. Other guidelines in this element address the need for multiple long and short term purposes and the definition of framework to ensure consistency and coherence of the assessment programme. The challenge in designing a programme of assessment will be to combine these different purposes in such a way that they are achieved in the optimal way with a clear hierarchy defined in terms of importance. This group of guidelines is aimed at supporting this combination.

Whereas in the original model *stakeholders* and *infrastructure* had been addressed last, they are now considered to be essential in many design decisions and are now considered at an early stage as well. Also, during the discussions, many guidelines led to questions about the organization and infrastructure, and the people needing to be involved. It was decided that it is imperative to establish parameters in relation to infrastructure, logistics, and staffing as soon as possible.

A4 *Opportunities as well as restrictions for the assessment programme should be identified at an early stage and taken into account in the design process.*

A7 *The level at which various stakeholders participate in the design process should be based on the purpose of the programme as well as the needs of the stakeholders themselves.*

### Programme in action

Since the key assessment activities are within this layer, it is no surprise that many of the guidelines relate to this aspect. Hence, most guidelines are about *collecting information*, especially the element that deals with selecting an assessment component. In line with general guideline (II), a rationale for the selection of instruments should be provided, preferably based on scientific research and/or best practice. The rationale should justify how components contribute to achieving the purpose of the assessment programme.

B1 *When selecting an assessment component for the programme, the extent to which it contributes to the purpose(s) of the assessment programme should be the guiding principle.*

During the interviews the experts agreed without much debate on the majority of guidelines about *collecting information* (B2-B9). These should aid in demonstrating the underpinning of the selection choices. Different components have different strengths and weaknesses and these have to be weighed against each other in order to decide the optimal balance to contribute to the purpose of the assessment. The interrelatedness of the guidelines should be taken into account in the design, but feasibility (Infrastructure) and acceptability (Stakeholders) are also clearly important. This is not as obvious as it seems. Currently design is often focussed almost exclusively on the characteristics of individual assessment components and not on the way in which they contribute to the programme as a whole. Often there is a tendency to evaluate the properties of an assessment component per se and not as a building block in the whole programme.

Around the guidelines about *combining information* there was considerably more discussion, therefore we decided to formulate them more generically and provide more elaborate explanations. Important within this group of guidelines is an underpinning for combing information (general guideline II), whereas in practice data is often combined based in similarity in format. (e.g. the results a communication station and a resuscitation station in one OSCE).

B14 *Combination of the information obtained by different assessment components should be justified based on meaningful entities either defined by purpose, content, or data patterns.*

Guidelines on *valuing information* and on *taking action* both consider the consequences (e.g. side effects) of doing so. Also links with other elements are explicitly made in these groups of guidelines.

B21 *Information should be provided optimally in relation to the purpose of the assessment to the relevant stakeholders.*

### Supporting the programme

In this layer, we found extensive agreement among the authors. Within the guidelines on *construction support*, next to the definition of tasks and procedures for support, special attention was given to faculty development as a supporting task as part of the availability of expertise to perform a certain task (general guideline III).

C4 *Support for constructing the assessment components requires domain expertise and assessment expertise.*

Guidelines on *political and legal support* are strongly related to the proportionality principle (general guideline I) and address procedures surrounding assessment, such as possibilities for appeal. This relates to seeking acceptance for the programme and acceptance of change which forms a basis for and links with *improving the programme*.

C6 *The higher the stakes, the more robust the procedures should be.*

C8 *Acceptance of the programme should be widely sought.*

### Documenting the programme

The fact that *rules and regulations* have to be documented did not raise much debate. These guidelines address the aspects that are relevant when considering the rules and regulations including the need for an organisational body, upholding the rules and regulations. The fact that the *context (e.g. learning environment)* in which the programme of assessment exists must be made explicit was self apparent.

A group of guidelines which received special attention in the discussions addressed *Domain Mapping*. The term blueprinting is deliberately not used here, because this term is often used to denote a specific tool using a matrix format to map the domain (content) to the programme and the instruments to be used in the programme. With Domain Mapping, a more generalised approach is implied. Not only should content match with components, but the focus should be on the assessment programme as a whole in relation to the overarching structure (e.g. the educational curriculum) and the purpose.

D9 *A domain map should be the optimal representation of the domain in the programme of assessment.*

### Improving the programme

The wording in this layer turned out to evoke different connotations. R&D in particular is defined differently in different assessment cultures. We therefore agreed to define r*esearch* in R&D as the systematic collection of all necessary information to establish a careful evaluation (critical appraisal) of the programme with the intent of revealing areas of strengths and areas for improvement. *Development* should then be interpreted as re-design. Once this shared terminology was reached, consensus on the guidelines came naturally.

E1 *A regular and recurrent process of evaluation and improvement should be in place, closing the feedback loop.*

Apart from measures to solve problems in a programme, political change or new scientific insights can also trigger improvement. *Change management* refers to activities to cope with potential resistance to change. (Political) acceptance of changes refers to changes in (parts of) the programme. Also these guidelines are related to the *political and legal support*.

E4 *Momentum for change has to be seized or has to be created by providing the necessary priority or external pressure.*

### Justifying the programme

The guidelines in this layer are more general, probably due to the fact that they are tightly related to the specific context in which a programme of assessment is embedded. Outcomes of good scientific research on assessment activities are needed to support assessment practices with trustworthy evidence, much like the drive for evidence-based medicine. Although this is a general principle which should guide the design of the programme as a whole, the guidelines about *effectiveness* become specifically important when one has to justify choices made in the programme.

F2 *New initiatives (developments) should be accompanied by evaluation, preferably scientific research.*

Guidelines on *cost-effectiveness* appear obvious as it is generally regarded as a desirable endeavour from a fit-for-purpose perspective. In every institution or organisation, resources - including those for assessment programmes - are limited. If the programme of assessment can be made more efficient, resources can be freed up for other activities. However, guidelines on this are rarely made explicit.

F6 *A cost-benefit analysis should be made regularly in light of the purposes of the programme. In the long term, a proactive approach to search for more resource-efficient alternatives should be adopted.*

The guidelines on *acceptability* are related to the issue of due practice. As an assessment programme does not exist within a vacuum, political and legal requirements often determine how the programme of assessment is designed and justified. An issue not often addressed during the design process is the use of outcomes by others, and related unintended consequences thereof.

F10 *Confidentiality and security of information should be guaranteed at an appropriate level.*

## Discussion and conclusion

We developed a comprehensive set of guidelines for designing programmes of assessment. Our aim was to formulate guidelines that are general enough to be applicable to a variety of contexts. At the same time they should be sufficiently meaningful and concrete as to support assessment designers. Since we tried to keep away from specific contexts or educational approaches, it is likely that this set may be applicable beyond the domain of medical education. Although these guidelines are more general than existing sets of guidelines, criteria or standards, we cannot dismiss that our backgrounds (i.e. medical education) might have resulted in too restrictive formulations of guidelines. This stresses the need for further replication of our study and on application of these guidelines in a range of contexts.

Although establishing guidelines is an ongoing process, it is remarkable that in a short time such a good consensus was reached among the experts. Most of the debate actually focused around a few specific guidelines, probably those that are more difficult to enunciate or less certain in their utility. For example topics like *combining information* remain still highly debated, and no complete and final answers can be provided at this time.

In trying to be as comprehensive as possible we acknowledge the risk of being over-inclusive. We would like to stress that when designing a programme of assessment, these guidelines should be applied with caution. We recognise and indeed stress that contexts differ and not all guidelines may be relevant in all circumstances. Hence, designing an assessment programme implies making deliberate choices and compromises, including the choice of which guidelines should take precedence over others. Nevertheless, we feel this set combined with the framework of programmes of assessment enables designers to keep an overview of the complex dynamics of a programme of assessment. An interrelated set of guidelines aids designers in foreseeing problematic areas, which otherwise would remain implicit until real problems arise.

We must stress that the guidelines do not replace the need for assessment expertise. Hence, given our fitness-for-purpose perspective on quality, putting the challenge in applying these general guidelines to a local context. Such a translation from theory into practice is not easy and we see the possibility of providing a universally applicable prescriptive design plan for assessment programmes to be slim. Only, if a specific purpose or set of purposes could be decided upon, one could argue that a set of guidelines could be prescriptive. However, thus far it has been the experience that one similar purpose across contexts is extremely rarely found, let alone a similar set of purposes.

What our guidelines do not support is how to make decisions, but they stress the need for decisions to be underpinned and preferably based on solid evidence. This challenge also provides an opportunity to learn from practice. Different ways of applying the guidelines will likely result in more sophisticated guidelines, and provide a clearer picture of the relations in the framework. Thus, it is probably inevitable that some guidelines are not self-evident and need more explanation. Real-life examples from different domains or educational levels will be required to provide additional clarity and understanding. This is a longer term endeavour beyond the scope of this paper. Also, it will involve more data gathering and examples from various domains.

Although validation by the opinions of experts is susceptible to biases, it was suitable in our study for generating a first concrete set of guidelines. The validation at this stage is divergent in nature and therefore inclusive and, as such, the guidelines might be over-inclusive. This is only one form of validation and not all guidelines can be substantiated with scientific evidence or best practice. Therefore further validation through specific research is necessary, especially in the area of implementation and translation to practice. Different programmes of assessment will have to be analysed in order to determine whether the guidelines are useful in practice and are generally applicable in different contexts. A practical validation study is now needed. It is encouraging to have already encountered descriptions of programmes of assessment in which to some extent the guidelines are intuitively or implicitly appreciated and taken into account. Of course this is to be expected since not all guidelines are new. However, we think that the merit of this study is the attempt to provide a comprehensive and coherent listing of such guidelines.

## Competing interests

The authors declare that they have no competing interests.

## Authors’ contributions

JD, CVDV and LWTS started with a brainstorm phase to generate the initial set (based on previous discussions) followed by a series of discussions with all authors. JD reformulated the guidelines. Peer debriefing was done to check the reformulation by JD, CvdV, & LWTS. JD checked with each author to reach consensus. All authors provided feedback on the manuscript, and read and approved the final manuscript.

## Pre-publication history

The pre-publication history for this paper can be accessed here:

http://www.biomedcentral.com/1472-6920/12/20/prepub

## Supplementary Material

Additional file 1 **Addendum complete set of guidelines - BMC Med Educ - final.doc.** This addendum contains the set of 72 guidelines developed and validated in this study. Click here for file

## References

[B1] Lew SR Page GG Schuwirth LWT Baron-Maldonado M Lescop JMJ Paget NS Southgate LJ Wade WB Procedures for establishing defensible programmes for assessing practice performance Medical Education 2002 36 936 941 10.1046/j.1365-2923.2002.01319.x 12390461

[B2] Schuwirth LWT Southgate L Page GG Paget NS Lescop JMJ Lew SR Wade WB Baron-Maldonado M When enough is enough: a conceptual basis for fair and defensible practice performance assessment Medical Education 2002 36 925 930 10.1046/j.1365-2923.2002.01313.x 12390459

[B3] Van der Vleuten C Schuwirth LWT Assessing professional competence: from methods to programmes Medical Education 2005 39 309 317 10.1111/j.1365-2929.2005.02094.x 15733167

[B4] Savage JK In-training assessment (ITA): designing the whole to be greater than the sum of the parts Medical Education 2006 40 13 16 10.1111/j.1365-2929.2005.02377.x 16441317

[B5] Dijkstra J Van der Vleuten C Schuwirth L A new framework for designing programmes of assessment Adv Heal Sci Educ 2010 15 379 393 10.1007/s10459-009-9205-z PMC2940030 19821042

[B6] Dannefer EF Henson LC The Portfolio Approach to Competency-Based Assessment at the Cleveland Clinic Lerner College of Medicine Academic Medicine 2007 82 493 502 10.1097/ACM.0b013e31803ead30 17457074

[B7] Davies H Archer J Southgate L Norcini J Initial evaluation of the first year of the Foundation Assessment Programme Medical Education 2009 43 74 81 10.1111/j.1365-2923.2008.03249.x 19141000

[B8] Ricketts C Bligh J Developing a Frequent Look and Rapid Remediation Assessment System for a New Medical School Academic Medicine 2011 86 67 71 10.1097/ACM.0b013e3181ff9ca3 21099391

[B9] Birenbaum M Evaluating The Assessment: Sources Of Evidence For Quality Assurance Studies in Educational Evaluation 2007 33 29 49 10.1016/j.stueduc.2007.01.004

[B10] Burch V Norman G Schmidt H Van der Vleuten C Are specialist certification examinations a reliable measure of physician competence? Adv Heal Sci Educ 2008 13 521 533 10.1007/s10459-007-9063-5 17476579

[B11] Harlen W Criteria for evaluating systems for student assessment Studies in Educational Evaluation 2007 33 15 28 10.1016/j.stueduc.2007.01.003

[B12] Knight PT The Value of a Programme-wide Approach to Assessment Assessment & Evaluation in Higher Education 2000 25 237 251 10.1080/71361143422900245

[B13] Wass V McGibbon D Van der Vleuten C Composite undergraduate clinical examinations: how should the components be combined to maximize reliability? Medical Education 2001 35 326 330 10.1046/j.1365-2923.2001.00929.x 11318994

[B14] Biggs J Enhancing teaching through constructive alignment High Educ 1996 32 347 364 10.1007/BF00138871

[B15] Cilliers F Schuwirth L Adendorff H Herman N van der Vleuten C The mechanism of impact of summative assessment on medical students’ learning Adv Heal Sci Educ 2010 15 695 715 10.1007/s10459-010-9232-9 PMC2995206 20455078

[B16] Cilliers F Schuwirth L Herman N Adendorff H van der Vleuten C A model of the pre-assessment learning effects of summative assessment in medical education Advances in Health Sciences Education 2011 online first 10.1007/s10459-011-9292-5 PMC3274672 21461880

[B17] Baartman LK Assessing the assessment: Development and use of quality criteria for competence assessment programmes 2008 Universiteit Utrecht,

[B18] ERA, APA, NCME Standards for Educational and Psychological Testing 1999 AERA, Washington

[B19] Schuwirth LWT Van der Vleuten CPM Programmatic assessment: From assessment of learning to assessment for learning Medical Teacher 2011 33 478 485 10.3109/0142159X.2011.565828 21609177

[B20] Harvey L Green D Defining Quality Assessment & Evaluation in Higher Education 1993 18 9 34 10.1080/026029393018010222900245

