# Supplementary material for: Expert validation of fit-for-purpose guidelines for designing programmes of assessment
Source: BMC Med Educ. 2012 Apr 17;12:20. doi: 10.1186/1472-6920-12-20 (PMC3676146; doi:10.1186/1472-6920-12-20)
Supplement: Additional file 1 — Addendum complete set of guidelines - BMC Med Educ - final.doc. This addendum contains the set of 72 guidelines developed and validated in this study. [file 1472-6920-12-20-S1.docx]

# Addendum: Guidelines for Designing Programmes of Assessment

# Table of content

**Introduction**

**GENERAL GUIDELINES**

I Decisions (and their consequences) should be proportionate to the quality of the information on which they are based.

II Every decision in the design process should be underpinned preferably supported by scientific evidence or evidence of best practice. If evidence is unavailable to support the choices made when designing the programme of assessment, the decisions should be identified as high priority for research.

III Specific expertise should be available (or sought) to perform the activities in the programme of assessment.

**PURPOSE OF THE PROGRAMME**

A1 One principal purpose of the assessment programme should be formulated.

A2 Long-term and short-term purposes should be formulated. But the number of purposes should be limited.

A3 An overarching structure which projects the domain onto the assessment programme should be constructed.

**INFRASTRUCTURE**

A4 Opportunities as well as restrictions for the assessment programme should be identified at an early stage and taken into account in the design process.

A5 Design decisions should be checked against consequences for the infrastructure. If necessary compromises should be made, either adjusting the purpose(s) of the assessment programme or adapting the infrastructure.

**STAKEHOLDERS**

A6 Stakeholders of the assessment programme should be identified and a rationale provided for including the expertise of different stakeholders (or not) and the specific role(s) which they should fulfil.

A7 The level at which various stakeholders participate in the design process should be based on the purpose of the programme as well as the needs of the stakeholders themselves.

**PROGRAMME IN ACTION**

**Collecting Information**

B1 When selecting an assessment component for the programme, the extent to which it contributes to the purpose(s) of the assessment programme should be the guiding principle.

B2 When selecting an assessment (component or combination), consideration of the content (stimulus) should take precedence over the response format.

B3 The assessment should sample the intended cognitive, behavioural or affective processes at the intended level.

B4 The information collected should be sufficiently informative (enough detail) to contribute to the purpose of the assessment programme.

B5 The assessment should be able to provide sufficient information to reach the desired level of certainty about the contingent action.

B6 The effect of the instruments on assessee behaviour should be taken into account.

B7 The relation between different assessment components should be taken into account

B8 The overt and covert costs of the assessment components should be taken into account and compared to alternatives.

B9 Assessment approaches that work well in a specific context (setting) should first be re-evaluated before use in another context (setting) before implementation.

B10 A programme of assessment should deal with error and bias in the collection of information. Error (random) is unpredictable and should be reduced by sampling (strategies). Bias (Systematic) should be analysed and its influence should be reduced by appropriate measures.

B11 Any performance categorisation system should be as simple as possible.

B12 When administering an assessment (component), the conditions (time, place, etc.) and the tasks (difficulty, complexity, authenticity, etc) should support the purpose of the specific assessment component.

B13 When scheduling assessment, the planning should support instruction and provide sufficient opportunity for learning.

**Combining Information**

B14 Combination of the information obtained by different assessment components should be justified based on meaningful entities either defined by purpose, content, or data patterns.

B15 The measurement level of the information should not be changed.

B16 The consequences of combining information obtained by different assessment components, for all stakeholders, should be checked.

**Valuing Information**

B17 The amount and quality of information on which a decision is based should be in proportion to the stakes.

B18 A rationale should be provided for the standard setting procedures.

**Taking Action**

B19 Consequences should be proportionally and conceptually related to the purpose of the assessment and justification for the consequences should be provided.

B20 The accessibility of information (feedback) to stakeholders involved should be defined.

B21 Information should be provided optimally in relation to the purpose of the assessment to the relevant stakeholders.

**SUPPORTING THE PROGRAMME**

**Construction Support**

C1 Appropriate central governance of the programme of assessment should be in place to align different assessment components and activities.

C2 Assessment development should be supported by quality review to optimise the current situation (Programme in Action), appropriate to the importance of the assessment.

C3 The current assessment (Programme in Action) should be routinely monitored on quality criteria.

C4 Support for constructing the assessment components requires domain expertise and assessment expertise.

C5 Support tasks should be well-defined and responsibilities should lie with the right persons.

**Political and Legal Support**

C6 The higher the stakes, the more robust the procedures should be.

C7 Procedures should be made transparent to all stakeholders.

C8 Acceptance of the programme should be widely sought.

C9 Protocols and procedures should be in place to support appeal and second opinion.

C10 A body of appeal should be in place

C11 Safety net procedures should be in place to protect both assessor and assessee.

C12 Protocols should be in place to check (the programme in action) on proportionality of actions taken and carefulness of assessment activities.

**DOCUMENTING THE PROGRAMME**

**Rules and Regulations (R&R)**

D1 Rules and regulations should be documented.

D2 Rules and regulations should support the purposes of the programme of assessment.

D3 The impact of rules and regulations should be checked against managerial, educational, and legal consequences.

D4 In drawing up rules and regulations one should be pragmatic and concise, to keep them manageable and avoid complexity.

D5 R&R should be based on routine practices and not on incidents or occasional problems.

D6 There should be an organisational body in place to uphold the rules and regulations and take decisions in unforeseen circumstances.

**Learning Environment**

D7 The environment or context in which the assessment programme has to function should be described.

D8 The relation between educational system and assessment programme should be specified.

**Domain Mapping**

D9 A domain map should be the optimal representation of the domain in the programme of assessment.

D10 A domain map should not be too detailed.

D11 Starting point for a domain map should be the domain or content and not the assessment component.

D12 A domain map should be a dynamic tool, and as a result should be revised periodically.

**IMPROVING THE PROGRAMME**

**R&D**

E1 A regular and recurrent process of evaluation and improvement should be in place, closing the feedback loop.

E2 If there is uncertainty about the evaluation, more information about the programme should be collected.

E3 In developing the programme (re-design) again improvements should be supported by scientific evidence or evidence of best practice.

**Change Management**

E4 Momentum for change has to be seized or has to be created by providing the necessary priority or external pressure.

E5 Underlying needs of stakeholders should be made explicit.

E6 Sufficient expertise about change management and about the local context should be sought.

E7 Faculty should be supported to cope with the change by providing adequate training

**JUSTIFYING THE PROGRAMME**

**Effectiveness**

***Scientific Research***

F1 Before the programme of assessment is designed, evidence should to be reviewed.

F2 New initiatives (developments) should be accompanied by evaluation, preferably scientific research.

***External Review***

F3 The programme of assessment should be reviewed periodically by a panel of experts.

F4 Benchmarking against similar assessment programmes (or institutes with similar purposes) should be conducted to judge the quality of the programme.

**Efficiency: *cost-effectiveness***

F5 In order to be able to justify the resources used for the assessment programme, all costs (in terms of resources) should be made explicit.

F6 A cost-benefit analysis should be made regularly in light of the purposes of the programme. In the long term, a proactive approach to search for more resource-efficient alternatives should be adopted.

**Acceptability: *political-legal justification***

F7 Open and transparent governance of the assessment programme should be in place and can be held accountable

F8 In order to establish a defensible programme of assessment there should be one vision (on assessment) communicated to external parties.

F9 The assessment programme should take into account superseding legal frameworks.

F10 Confidentiality and security of information should be guaranteed at an appropriate level.

# Introduction

The authors present these guidelines to be read with the following points in mind.

1. There is no linear order in the guidelines presented.

When reading the guidelines, you may not immediately come across those guidelines or important topics you would expect to be given priority. There is potentially more than one way of ordering the guidelines. As one example *costs* are important throughout the design process. However, because of the way this framework is constructed, *costs* are addressed near to the end.

1. There is overlap between guidelines.

It appeared impractical and somewhat artificial to split every assessment activity into separate parts. The guidelines are highly related, and overlap and/or redundancy are almost inevitable. In the example of *costs*, which are primarily addressed as part of *cost-efficiency*, references to *costs* are actually made in several guidelines.

1. The level of granularity is not equal for all guidelines.

Determining the right level of detail is a difficult endeavour, variable granularity reflects the fact that some issues seem more important than others, and others may have been investigated in depth.

1. Assessment components and assessment information.

In the guidelines we have sought to find an overarching term that would cover all possible elements of the programme, such as assessments, tests, examinations, feedback, and dossiers. We wanted the guidelines to be broadly applicable, and so we have chosen the term assessment *components*. Similarly for outcomes of assessment components we have chosen assessment *information* (e.g. data about the assessees’ competence or ability).

<<<INSERT FIGURE 1 ABOUT HERE>>>

Figure 1: Framework

# GENERAL GUIDELINES

Three major themes emerged and are set out below. They are general and applicable to the design process as a whole. Although these guidelines are formulated more generally at this point, we will refer to these explicitly again in the separate layers as a reminder.

#### I Decisions (and their consequences) should be proportionate to the quality of the information on which they are based.

This guideline has implications for all aspects of the assessment programme, both at the level of the design of the programme, and at the level of individual decisions about assessees’ progress. The higher the stakes, the more robust the information needs to be. In the layer *Programme in Action* for instance, actions based on (collected) information should be proportionate to the quantity and quality of the information. The more high-stakes an action or decision, the more certainty (justification and accountability) is required, the more the information collection process has to comply with scientific criteria, and usually the more information that is required. If the subsequent action means that a assessee has to retake one examination, it has less impact when the action means the assessee has to retake an entire year of medical school. Therefore, the former can be taken on the basis of less information (e.g. the results of one single test). The latter, however, requires a series of assessments or maybe even a dossier.

II Every decision in the design process should be underpinned preferably supported by scientific evidence or evidence of best practice. If evidence is unavailable to support the choices made when designing the programme of assessment, the decisions should be identified as high priority for research.

This implies that all choices made in the design process should be defensible and can be justified. Even if there is no available scientific evidence, a plausible or reasonable rationale should be proposed. Evidence can be sought through a survey of the existing literature, new research endeavours, collaborative research, or even be outsourced completely. We stress again that the fitness-for-purpose principle should guide design decisions, i.e. which decisions will contribute optimally to achieving the purpose(s).

III Specific expertise should be available (or sought) to perform the activities in the programme of assessment.

This guideline is more specifically aimed at the expertise needed for the assessment activities in the separate layers and elements within the assessment programme. The challenge in setting up a programme of assessment is to “get the right person for the right job”. Expertise is often needed from different fields including assessment expertise. Legal expertise, specific domain or content knowledge, and practical knowledge about the organisation are frequently required. Some types of expertise, such as psychometric expertise for item analysis, and legal expertise for rules and regulations, are obvious. Others are less clear and more context specific. It is useful when designing an assessment programme to articulate the skill set and the body of knowledge that is useful or even necessary to address these issues.

# PURPOSE OF THE PROGRAMME

***Purpose of the Programme*** constitutes a central role in the model for programmes of assessment. It is impossible to consider other assessment elements in isolation from the purpose. Regardless of educational approach (e.g. lecture-based education, problem-based learning) or the specific function of assessment (e.g. learning tool, licensing decisions), the quality of assessment programmes should be framed in terms of ***fitness-for-purpose***.

A1 One principal purpose of the assessment programme should be formulated.

This principal purpose should contain the function of the assessment programme and the domains to be assessed. The principal purpose should be formulated by high level stakeholders within the organisation, who are able to oversee the big picture and who understand the context which the organisation has to deal with. In many cases a programme of assessment has to take into account (and contribute to) multiple purposes. Defining one principal purpose of the programme might seem too ideal and theoretical, as the real world is messy. However, defining a principal purpose should contribute to coherence and consistency of the programme as a whole. E.g. in case of conflicts of interest a principal purpose should provide guidance for deciding on compromises. The challenge in designing a programme of assessment will be to combine these different purposes in such a way that they are achieved in the optimal way with a clear hierarchy defined in terms of importance.

A2 Long-term and short-term purposes should be formulated. But the number of purposes should be limited.

Although intuitively one would think that defining one principal purpose is ideal, such purposes are often defined to vaguely or too restrictive. Therefore in this guideline we advise to formulate short term (sub) purposes that will define more concretely the road map to achieving the main long term purpose. More than one purpose may be imposed by the dynamics of the environment as well. Not all purposes may come from within the organisation; external stakeholders might also exert influence. Defining these purposes enables their constructive inclusion in planning the programme of assessment.

(A) Purposes should be made concrete and feasible, but also transparent and referable. (B) Purposes should be prioritised based on (among other things) the principal purpose. (C) Purposes and their prioritizing should be justified based on sufficient information (such as: literature and scientific research, stakeholders, educational approach, etcetera). Explanation of these sub purposes should be helpful in managing different stakes.

A3 An overarching structure which projects the domain onto the assessment programme should be constructed.

An overarching structure should provide the big picture of the assessment programme that needs to be designed. *Domain* in this guideline can be interpreted in the broadest sense of the word. The overarching structure should function as a framework to ensure consistency and coherence of the assessment programme. It has to be formulated with high-level descriptors instead of detailed specifications of items on a test. A more detailed description or map of the domain onto the programme has to be documented (see *Documenting the Programme*: *Domain Mapping*). There is no generally applicable overarching structure and existing structures have to be evaluated on a case by case basis.

E.g. the contemporary idea of competency-based education generally uses a series of competencies to structure the domain (e.g. CanMeds [1], Good Medical Practice [2], or ACGME[3]), although a simple list of topics to be covered could work just as well. From a different perspective, Miller’s pyramid [4] dividing the domain into aspects of competence (knows, knows how, shows how, does), can also be used to map the domain onto the assessment programme. Another example is the (instructional) curriculum (what is taught, when and where) defining the development of mastery of the domain by learners. When deciding on the overarching framework to use, it might be acceptable to combine different existing structures and select the appropriate aspects from these. However, content is not the overarching structure, it populates it.

# INFRASTRUCTURE

The layer *Infrastructure* deals with the physical and practical systems and structures an organisation needs to have in place to support a functional assessment programme (e.g. an administration office or logistics of assessment). This is in contrast to the element *Learning Environment* in the *Documenting* layer which describes more intangible aspects such as the culture of the organisation or institution and the educational setting or approach.

#### A4 Opportunities as well as restrictions for the assessment programme should be identified at an early stage and taken into account in the design process.

This guideline informs decisions regarding compromises on the purpose and /or resources (see A5). On a proactive note it is important to accept that it may not be possible to achieve all of the assessment ideals. Knowing the restrictions at an early stage prevents disappointment during the design phase and potential challenge during implementation.

#### A5 Design decisions should be checked against consequences for the infrastructure. If necessary compromises should be made, either adjusting the purpose(s) of the assessment programme or adapting the infrastructure.

Depending on the resources, urgency, and need to achieve certain purposes a balance has to be found between investing in infrastructure and making concessions to the purpose. Expertise in administrating tests and in logistics of the organisation is necessary. E.g. deciding to implement computerised examinations might simplify logistics of administrating tests and calculating scores. However, this decision has resource implication and may put a strain upon IT support and computer/network-facilities.

# STAKEHOLDERS

Stakeholders are inextricably part of the programme as well as the design process. Although in various elements in the model, stakeholders are mentioned (as in *Acceptability* in the layer *Supporting the Programme*), it is important to mention some aspects separately.

#### A6 Stakeholders of the assessment programme should be identified and a rationale provided for including the expertise of different stakeholders (or not) and the specific role(s) which they should fulfil.

This enables an informed decision to be made based on the involvement of stakeholders (in what phase or in what element). The roles they should play in the design and/or assessment programme should be clarified. Different perspectives can be distinguished, in relation to which several subgroups can be defined.

1. **society** (customers): e.g., patients, medical councils, government, tax-payers.
2. **assessee** (product): student, candidate, learner
3. **faculty** (company): management, teachers.

#### A7 The level at which various stakeholders participate in the design process should be based on the purpose of the programme as well as the needs of the stakeholders themselves.

The purpose of the assessment programme might render participation of some groups unnecessary or require that other groups participate. In each instance, a case should be made to demonstrate how involvement supports the principal purpose.

# PROGRAMME IN ACTION

*Programme in Action* defines the currently running assessment practices. The four core activities of *Programme in Action* are: *Collecting Information*, *Combining Information*, *Valuing Information* and *Taking Action*. This layer includes the activities minimally required to have a running assessment programme. As such, this is a necessary but not sufficient condition for a high-quality programme. This layer encompasses activities ranging from collecting information to taking action based on that information.

## COLLECTING INFORMATION

#### B1 When selecting an assessment component for the programme, the extent to which it contributes to the purpose(s) of the assessment programme should be the guiding principle.

In line with general guideline (II), a rationale for the selection of assessment components should be provided, preferably based on scientific research and/or best practice. Equally, the contribution each component makes to achieve the purpose of the assessment programme should be considered.

The guidelines in this section (B2-B9) should aid in demonstrating the underpinning of the selection choices. Different components have different strengths and weaknesses and these have to be weighed against each other in order to decide the optimal balance to contribute to the purpose of the assessment. The interrelatedness of the guidelines should be taken into account in the design, but feasibility (*Infrastructure*) and acceptability (*Stakeholders*) are also clearly important. This is not as obvious as it seems. Currently design is often focussed almost exclusively on the characteristics of individual assessment components and not on the way in which they contribute to the programme as a whole. Often there is a tendency to evaluate the properties of an assessment component per se and not as a building block in the whole programme.

#### B2 When selecting an assessment (component or combination), consideration of the content (stimulus) should take precedence over the response format.

The target for assessment should determine the type of assessment components. A common pitfall arises because of the ready availability of assessment components or question formats. This results in fitting the question to the format. From research we know that the stimulus (content of the question) is more important than the format of the assessment. No response format is by definition better than another (form follows content). A similar format e.g. an open-ended question can be used to measure different types of knowledge e.g. Factual knowledge: “Who is the President of the United States?”; or Clinical reasoning: “Given the case described, what is the correct diagnosis? [5-8]

#### B3 The assessment should sample the intended cognitive, behavioural or affective processes at the intended level.

In addition to content, the mental, behavioural, and affective processes evoked in assessees should also support the purposes of the assessment. A good doctor has to have the ability to deal with a variety of situations. To assess this ability, a programme of assessment has to be constituted using a range of assessment components. Many, if not all, assessment programmes already use a mix to get a complete picture of assessee performance in the domain of interest.

#### B4 The information collected should be sufficiently informative (enough detail) to contribute to the purpose of the assessment programme.

The goal of this guideline is to ensure that the information collected in the assessment programme can be used to fulfil (one of) its purpose(s). When selecting assessment components the characteristics of the information should be considered in relation to the purposes. A pass/fail or yes/no could suffice to permit assessees to practise medicine. However, if the (sub) purpose is aimed at improvement of the assessee then further information is necessary to inform the assessee about how to improve. Different characteristics can be of importance under different circumstances; If the priority is to measure incremental change, multiple measurements over time are required. If a purpose is to measure improvement (e.g. of the assessee or the educational programme), the collected information should be comparable to previous measurements. If the aim is to compare results on different topics, the results should not be combined into one score on the assessment, but should provide information on each topic.

#### B5 The assessment should be able to provide sufficient information to reach the desired level of certainty about the contingent action.

This guideline is a specific instance of general guideline I to ensure that the information gathered is of sufficient quality to ensure that consequent actions are consistent with the strength of the information. The higher the stakes the more certainty that is required to come to a decision and act on it. Sufficient information pertains to the amount of information in relation to its reproducibility. This raises the question “When is enough enough?” [9]. It also works the other way around. If sufficient certainty exists that the intended decisions (action) will not change with additional information, collection of more information is not useful. But if the purpose cannot be achieved with sufficient certainty, further collection of information is necessary.

#### B6 The effect of the instruments on assessee behaviour should be taken into account.

Assessment drives learning. The assessment programme should support the educational principles or perspective on learning (if applicable) and contribute to the instruction (i.e. educational programme). Assessment should not hinder learning (or development), but it can be employed strategically to steer learning behaviour and thus strengthen the instructional value, e.g. Constructive Alignment [10].

#### B7 The relation between different assessment components should be taken into account

The goal of this guideline is to avoid competition between instruments, but also to achieve efficiency of the programme. The aim is to achieve an optimal mix of instruments, usually by using strengths of one instrument to compensate for weaknesses of others.

Selection of instruments should result in a balanced compromise. Redundancy of information can be reduced for efficiency reasons; or be fostered in order to triangulate data. In other words, when selecting a mix of instruments, the method of combining information has to be taken into account. Combining information can also influence learning behaviour. If the weighting is unbalanced, assessees tend to study harder for the test that has the most weight.

#### B8 The overt and covert costs of the assessment components should be taken into account and compared to alternatives.

*Costs* is a separate element in the *Justifying the Programme* dimension. However, we feel this is an important aspect in the selection of assessment components and also worth mentioning here.

#### B9 Assessment approaches that work well in a specific context (setting) should first be re-evaluated before use in another context (setting) before implementation.

This guideline refers to that fact that there are not many *assessment activities* that are generally applicable across contexts. Every context has its own issues with feasibility and validity of an assessment component. Although it can be seen as best practice in one situation, it might be less so in another setting. Applicability of the assessment in its own specific context should be considered.

#### B10 A programme of assessment should deal with error and bias in the collection of information. Error (random) is unpredictable and should be reduced by sampling (strategies). Bias (Systematic) should be analysed and its influence should be reduced by appropriate measures.

Error and bias in assessment are unavoidable and must be taken into account when designing an assessment programme.

Error is random: A single measurement or data-point is always flawed as a result of unsystematic error. More samples (as measurements or data-points) are needed to reduce the effect of error on the reliability of the assessment. Depending on the purpose and the stakes influencing the need for reliability, a trade-off decision should be made between efficiency and costs on the one hand and broad sampling on the other.

To deal with systematic bias, awareness of the bias has to be fostered and the source of the bias should be made transparent. Efforts to manage bias should be directed towards the source (e.g. the test material or the user).

Bias in the test material (e.g. in a written test) is best tackled by improving the material (structuring, reviewing items, etc.) Bias as a result of (human) judgement, is best tackled by professionalising (training) the assessor. This means that improving the quality of assessment which is based on observation requires effort to be focussed on the user (i.e. assessor) e.g. by training, or fostering acceptability.

#### B11 Any performance categorisation system should be as simple as possible.

A performance system should be as simple as possible e.g. complex scoring systems do not add value to the assessment information; more often than not they complicate the interpretation. There is a tendency to give more weight to key items (and constructing *killer*-items), in order to increase validity of assessment. However, this contradicts the need for many items to achieve reliability of assessment. Validity should not be addressed by scoring systems, but by constructing high quality items or increasing complexity of items. The decisions based on different scoring systems do not vary much [11]. Differences occur around the pass-fail decision.

#### B12 When administering an assessment (component), the conditions (time, place, etc.) and the tasks (difficulty, complexity, authenticity, etc) should support the purpose of the specific assessment component.

In different stages of the assessment programme (or curriculum) the conditions and tasks may vary in their characteristics. Unnecessary (cognitive) load should be avoided. The level of the assessee should be considered. The context in which competency should be demonstrated should be supported by the selection of the instrument.

#### B13 When scheduling assessment, the planning should support instruction and provide sufficient opportunity for learning.

Assessment drives learning and as such determines the focus of assessees. Planning an assessment component too close to the time of instruction might undermine the attention given to this instruction, as assessees might already focus on the assessment component.

Similarly people need time to study and to learn new things or to remediate deficiencies. Therefore sufficient time should be provided between instruction and assessment, as well as between assessment and re-sitting of the assessment.

A distinction can be made between longitudinal development versus a more ad hoc (just in time) development.

## COMBINING INFORMATION

#### B14 Combination of the information obtained by different assessment components should be justified based on meaningful entities either defined by purpose, content, or data patterns.

Different purposes (e.g. decisions regarding assessees versus decisions about the educational programme) require different ways of combining the information from the assessment components. This may involve approaches that do not necessarily combine results merely because they are of the same format (e.g. the results a communication station and a resuscitation station in one OSCE). What meaningful elements are is best defined in the overarching structure of the domain (guideline A3). To illustrate this further, if in patient care we would adopt the same standard procedure of combining information as in many assessment programmes, we would combine the sodium level and the potassium level because they are both of the same ‘format’. But this combination is less meaningful than the combination of the sodium level and e.g. complaints of thirst. Unfortunately, available research in this area is minimal. The question remains: How to combine information from various (qualitative and quantitative) sources in a more meaningful way and reach a decision. But the paucity of this research only supports its urgency.

#### B15 The measurement level of the information should not be changed.

The measurement level of the information should not be changed just to be able to add things up. Qualitative scores should not be converted into quantitative scores. Often qualitative information is more useful than quantitative information. Different kinds of evidence should be juxtaposed in some way or another, but not necessarily numerically just to allow averaging. Hence, combining information is not necessarily about reducing information, but can also be about finding similar messages in the information. Other ways of combining should be explored, such as holistic or global judgments, triangulation, emerging themes and other methods from qualitative research.

#### B16 The consequences of combining information obtained by different assessment components, for all stakeholders, should be checked.

Combining information can mean loss of information (data reduction). this can be simultaneously useful for some stakeholders and useless for others: knowing whether you passed or failed does not tell you anything about your strengths or weaknesses.

## VALUING INFORMATION

#### B17 The amount and quality of information on which a decision is based should be in proportion to the stakes.

This is a specification of general guideline I and guidelines B4 & B5. Whether enough information is collected depends among other things on the consequences of the actions to be taken based on this information. The higher the stakes, the more information is required to eliminate uncertainty in the outcome of the assessment e.g. failing an assessee from medical school based on one MCQ test is disproportionate.

#### B18 A rationale should be provided for the standard setting procedures.

This is also the underpinning of the set standard. The standard setting procedure should be chosen in light of to stakes, resources, and acceptability of false positives and false negatives. This is a specification of general guideline I. When stakes are high, justification of the standard setting procedures needs to be stronger to support the defensibility of standards. When the effect of incorrect decisions is severe, more care (effort and evidence) should be put into standard setting. In cases where human judges are assessing, they often use implicit standards. These should be made explicit and justified in order to achieve more defensible standards. Although often a decision has to be made as to what constitutes a pass, in the extreme case of a pure formative assessment, where the stakes are very low, a standard can be implicitly put in narrative feedback from an individual assessor to the assessee. Standard setting is always arbitrary but should never be capricious. Every standard contains more or less arbitrary decisions. To enhance acceptability and defensibility a rationale for these arbitrary decisions should be made explicit e.g. availability of a norm population, number of assessees, availability to provide a judgement, resources available, etc. In addition there is also the perspective on the standard that is set e.g. a statistical, an ethical, or psychological rationale for the standard.

## TAKING ACTION

#### B19 Consequences should be proportionally and conceptually related to the purpose of the assessment and justification for the consequences should be provided.

This is a specification of general guidelines I & II. Severe consequences should be based on extensive and high quality assessment, whereas minor consequences can be justified with less information or information of lower quality.

#### B20 The accessibility of information (feedback) to stakeholders involved should be defined.

Information should be accessible to the appropriate stakeholders. How much information is provided and to whom, depends on the purpose and context of the assessment.

#### B21 Information should be provided optimally in relation to the purpose of the assessment to the relevant stakeholders.

In order to have the desired effect, the information (based on the purpose of the assessment programme) should reach the right persons in the right manner. Therefore care has to be taken in determining how to present feedback in order to optimise the intended results (including sub purposes) of the assessment programme. In some cases this means extensive feedback to achieve a change in learning behaviour, whereas in other cases a simple pass-fail notification can be sufficient. Feedback needs to be moderated and annotated so that users or receivers can understand the information and how it was collected, instead of just dumping information on receivers. This also implies that expertise on how to provide information is required e.g. faculty development on giving feedback might be beneficiary.

# SUPPORTING THE PROGRAMME

*Supporting the Programme* includes activities contributing to the quality of the programme of assessment, which more often than not are related to, if not interwoven with, activities categorised under *Programme in Action*. This is about quality support activities, as distinct from infrastructural support. For an activity to support the programme in action and contribute to overall programme quality it should be directed at the purposes of the assessment programme. Supporting activities must ensure that the programme in action is of sufficient quality to contribute optimally to the purpose of the assessment programme. The following two support-related themes are congruent with the concept of quality as being fitness-for-purpose. Together with *Programme in Action*, *Supporting the Programme* forms a cyclic process aimed at optimising the internal assessment system.

## CONSTRUCTION SUPPORT

#### C1 Appropriate central governance of the programme of assessment should be in place to align different assessment components and activities.

One of the main problems in a decentralised assessment programme is the level of relatedness of assessment components and assessment activities. A central body of some kind should be in place to avoid sub-optimization and counterproductive initiatives within the programme of assessment.

#### C2 Assessment development should be supported by quality review to optimise the current situation (*Programme in Action*), appropriate to the importance of the assessment.

The development of all assessment components should include pre- and post- administration quality procedures. The amount of effort invested in quality review depends on the purposes of the assessment.

Pre- administration procedures can include peer review of items (written assessment) or assessor training (observational assessment). Post- administration procedures can include assessor performance evaluation or psychometric analysis of items.

#### C3 The current assessment (*Programme in Action*) should be routinely monitored on quality criteria.

Evaluative information should be collected and acted upon. Evaluative information should be fed back to the current assessment programme and fed forward to a redesign to improve the programme and prevent mistakes in the future . Psychometric analysis and user satisfaction can be part of the quality review.

#### C4 Support for constructing the assessment components requires domain expertise and assessment expertise.

Both types of expertise should be included here. Improvements can then be made on various important aspects e.g. in content, format or assessment design. From this perspective, faculty development is an important quality improvement measure to enhance expertise in assessment issues and, as such, faculty development supports a programme of assessment. It was also seen as an activity to support the development and evaluation of the programme of assessment. Furthermore, it also relates to acceptance of the assessment programme (C8).

#### C5 Support tasks should be well-defined and responsibilities should lie with the right persons.

Expertise is used in the right place and at the right level (e.g. experts should be involved in constructing items (content) and not ticking boxes on a highly structured form). At the same time tasks should be appointed to specific persons to guarantee that things get done. For the same reason administrative support should be available.

## POLITICAL AND LEGAL SUPPORT

#### C6 The higher the stakes, the more robust the procedures should be.

The procedures around the assessment programme should be robust and should be able to withstand legal challenge. There should be due process, meaning that assessment components and activities should be defensible. This does not mean all procedures should be explicit, standardised, or objective. Rather this implies that procedures should be acceptable to, or defensible for, all stakeholders. E.g. with high stakes examinations, it is more important to have safety net procedures in place as the consequences of the outcome are more severe and have a greater impact on the assessee. In contrast, if the purpose of an exam is only to provide feedback for an individual, the stakes are low and less attention can be given to procedures (more leniency can be permitted). This is a specification of the general guideline (I) that actions should be proportional to the quality of the information. With robust procedures the quality of information and/or the quality of decision-making can be increased.

#### C7 Procedures should be made transparent to all stakeholders.

Procedures should be made easy to understand. Complexity and exceptions should be avoided as much as possible.

#### C8 Acceptance of the programme should be widely sought.

As the outcome of the assessment programme often influences stakeholders, it is important that the stakeholders accept the assessment programme. Although accepting the assessment does not necessarily mean liking it, stakeholders should buy into the programme of assessment and the instruments used in it. Especially when the user determines the quality of the instrument (using a scoring form), users’ opinions and motivation are critical.

#### C9 Protocols and procedures should be in place to support appeal and second opinion.

When decisions are made, often some disagreement with the decision arises. Having protocols in place to deal with disagreement makes the defensibility of decisions stronger (if it holds up), contributes to acceptance of stakeholders, and may avoid legal challenge. Such protocols and procedures also constitute a safety net or quality assurance opportunity to identify and address mistakes in the programme of assessment.

#### C10 A body of appeal should be in place

Filing an appeal should be safe for the applicant. Consideration of the appeal should be sufficiently objective e.g. by establishing a body, which is independent of the organisation, to deal with this.

#### C11 Safety net procedures should be in place to protect both assessor and assessee.

To avoid perverse actions or decisions both the assessor and assessee should be able to voice their opinion without sanctions. On the one hand assessors should be protected and supported when having to make unfavourable decisions, like failing an assessee, without negative consequences in terms of extra work or litigation. On the other hand, assessees should also be protected from unfair practice in assessment.

#### C12 Protocols should be in place to check (the programme in action) on proportionality of actions taken and carefulness of assessment activities.

Alongside the robustness of the procedures, processes should be in place to guarantee the appropriateness of the activities conducted in the assessment programme. Where the other guidelines focus on the design of the programme, these protocols are intended to check for appropriateness of the current activities (active after the design process).

# DOCUMENTING THE PROGRAMME

*Documenting the Programme* serves two purposes. Firstly, documentation will facilitate learning of the organisation by allowing the cycle of optimising the programme in action to function properly. Secondly, it enhances the clarity and transparency of the programme. It is about explaining procedures in the programme and what is to be expected. In essence this should be public information.

## RULES AND REGULATIONS (R&R)

#### D1 Rules and regulations should be documented.

Procedures on which decisions are made should be made explicit. Without having these stated (explicitly) decisions might become arbitrary, ambiguous, or *ad hoc*. In order to make decisions defensible, the procedures on which the decisions are based need to be documented. When formulating R&R input from people who are knowledgeable about the assessment programme is required. Legal expertise can contribute to the clarity of the R&R. All stakeholders are impacted by R&R to some extent, although it assesses and assessors are likely to be most affected by the procedures underlying decisions. Although it is expected that R&R should be documented in virtually any programme of assessment, documentation can differ in degree and is likely to be dependent on the stakes involved.

#### D2 Rules and regulations should support the purposes of the programme of assessment.

Rules and regulations should not have unintended consequences on the outcomes of the assessment programme or the behaviour of stakeholders. Not only does *assessment drives learning*, assessment also procedures drive learning. For instance when offering many opportunities to pass a test (i.e. a lot of resit possibilities), it can become realistic for assessees to take a test without studying for it, because they might pass by chance. (This is also an opportunity to gain experience of test content before taking a resit.)

#### D3 The impact of rules and regulations should be checked against managerial, educational, and legal consequences.

The goal of this guideline is to avoid unwanted and unintended consequences of the rules and regulations e.g. overuse of resources of an institution. There should be congruence between R&R and requirements of management, educationalists, and legal staff. Therefore it is important to have expertise not only in drawing up rules and regulations, but also in awareness of the higher level implications in the organisation.

#### D4 In drawing up rules and regulations one should be pragmatic and concise, to keep them manageable and avoid complexity.

Rules and regulations should be formulated and made transparent, available, unambiguous, fair, and simple*.* It is important that R&R can be understood by all relevant stakeholders and proportional effort should be spent in making sure this is achieved. Involvement of stakeholders in the review of the R&R does contribute to achieving this guideline and has the benefit of fostering acceptance of the R&R at the same time.

#### D5 R&R should be based on routine practices and not on incidents or occasional problems.

The more rules and regulations, the more time is needed to maintain them. While trying to be comprehensive in all instances one can spend a disproportionate amount of effort in covering rare cases or cases that will never occur.

In practice often R&R slowly increase in scope and complexity in response to one off incidents. This will tend to decrease transparency and increase complexity. If the R&R are too specific or detailed the programme can become inflexible and incapable of dealing with unforeseen circumstances. Although incidents can be a trigger to review R&R, these rare instances should normally be covered by a general clause (e.g. “In circumstances that these Rules and Regulations do not foresee, the certifying committee has the final say”).

#### D6 There should be an organisational body in place to uphold the rules and regulations and take decisions in unforeseen circumstances.

The responsibility of upholding the R&R should be clearly defined and an appropriate mandate should be given to a separate body.

## LEARNING ENVIRONMENT

Guideline D7 is about the context of the assessment programme and guideline D8 refers to the educational approach underlying an assessment programme.

#### D7 The environment or context in which the assessment programme has to function should be described.

The goal of this guideline is to contribute to the feasibility of implementing a programme of assessment with long term sustainability. Knowing the specifics of your own context and making them explicit supports the transfer of scientific research and best practice to one’s own practice.

#### D8 The relation between educational system and assessment programme should be specified.

The goal of this guideline is to achieve a match between the assessment programme and the underlying assumptions regarding learning, instruction, and assessment. In order to have an assessment programme that can fulfil its purpose, it needs to be in line with the educational approach. In this sense it can be also applied to a non-educational organisation (e.g. a certifying body), as these organisations (consciously or unconsciously) also use educational paradigms. Knowing the educational approach can contribute to a more consistent and coherent programme of assessment.

## DOMAIN MAPPING

The term *blueprinting* is deliberately not used here, because this term is often used to denote a specific tool using a matrix format to map the domain (content) to the programme and the instruments to be used in the programme. With *Domain Mapping*, a more generalised approach is implied. Not only should content match with components, but the focus should be on the assessment programme as a whole in relation to the overarching structure (e.g. the educational curriculum) and the purpose.

#### D9 A domain map should be the optimal representation of the domain in the programme of assessment.

First of all, a domain map relates to the overarching structure (guideline A3). This domain map entails a more detailed specification of the overarching structure, including assessment components and content elements. A domain map is closely tied to the sampling of content and the strategies used (*Collecting Information*), and to combining information on specific content from different sources. This is related to the fact that a single instrument is never sufficient to claim that a particular domain is completely and validly assessed. A variety of assessment components is required, and these have to be mapped onto the domain. As such, *Domain Mapping* is part of the validation process and, in accordance to guideline B2, content prevails over format. Aspects to consider in describing the domain are the content (knowledge, skills, attitude), and the level of authenticity (simulated versus real). The programme should sample purposefully through content and levels of authenticity.

#### D10 A domain map should not be too detailed.

This guideline is formulated in order to avoid the pitfall of atomizing a programme of assessment into the smallest possible units of analysis. This would harm the integrative nature of a programmatic approach towards assessment. As such, a domain map should not contain too much detail, or too many dimensions (axes). Too much detail would diminish the degrees of freedom in assessment and frustrate the process of designing as well as administering the assessment programme.

#### D11 Starting point for a domain map should be the domain or content and not the assessment component.

In congruence with guideline B2 the assessment component (e.g. type or format) is a tool not a goal. The domain or content that should be measured is part of the goal. Often an assessment component is available or familiar to the user or designer and therefore becomes the first choice when designing an assessment programme. In some cases there will be a sound match between content and instrument, however, this is not guaranteed. Starting from the purpose and the nature of the domain to be assessed will focus the design process on achieving the purpose.

#### D12 A domain map should be a dynamic tool, and as a result should be revised periodically.

There is a risk that the domain map quickly becomes outdated, as virtually every field develops at a rapid pace. Also priorities and ideas change over time. Therefore the domain map should be updated periodically. The frequency with which updating is needed, depends on the context of the assessment programme and the pace of developments in the domain.

# IMPROVING THE PROGRAMME

Activities related to *Improving the Programme* generally have no immediate effect on the currently running programme, but impact in the (re)design of (parts of) the programme, usually at a later date.

## R&D

Scientific research is dealt with in the next layer (*Justifying the Programme*). *Research* in R&D is defined as the systematic collection of all necessary information to establish a careful evaluation (critical appraisal) of the programme with the intent of revealing areas of strengths and areas for improvement. *Development* should then be interpreted as re-design and therefore all other guidelines apply.

#### E1 A regular and recurrent process of evaluation and improvement should be in place, closing the feedback loop.

Not only should information be collected about the functioning of the programme, it should be acted upon as well (e.g. plan-do-check-act).

#### E2 If there is uncertainty about the evaluation, more information about the programme should be collected.

This is a specification of general guideline I. Actions based on evaluation of the programme (development) more often than not have large implications for the organisation. Therefore before changes are implemented based on the evaluation information, a high level of certainty about the information is required.

#### E3 In developing the programme (re-design) again improvements should be supported by scientific evidence or evidence of best practice.

This is a specification of general guideline II. In a sense all guidelines are applicable in the case of re-design.

## CHANGE MANAGEMENT

Apart from measures to solve problems in a programme, political change or new scientific insights can also trigger improvement. *Change Management* refers to activities designed to cope with potential resistance to change. (Political) acceptance of changes refers to changes in (parts of) the programme.

#### E4 Momentum for change has to be seized or has to be created by providing the necessary priority or external pressure.

It is likely that many stakeholders do not perceive or experience the same imperative for change. The need for change has to be communicated or awareness should be raised to diminish possible resistance to change. The sense of urgency can be influenced by e.g. leadership, external pressure, and time, but also by making sure that stakeholders understand the reason(s) for change and how they can benefit from it. Often resistance to change stems from uncertainty and anxiety. Making the reasons for change explicit and communicating the benefits to stakeholders often decreases resistance. A change which at first sight is unpopular, may be accepted if it contributes sufficiently to the needs of the stakeholders.

#### E5 Underlying needs of stakeholders should be made explicit.

Because the *wants* of different stakeholders may seem to compete at first sight, the underlying needs of stakeholders should be made explicit. Needs may not be clear to the stakeholders themselves as they are often less noticeable than wants. Wants can concern a specific lay-out of a form, whereas the need might be that the form can be filled out quickly.

#### E6 Sufficient expertise about change management and about the local context should be sought.

Similar to *Construction Support*, there is a need to translate general concepts to the local situation. In assessment practices such as item writing the same issue occurs. One cannot write a test item without content expertise, nor without expertise on writing an item. Both expertises have to be combined in order to develop good items.

#### E7 Faculty should be supported to cope with the change by providing adequate training

Uncertainty and low efficacy lead to resistance. Clear explanations and introductions as well as training or faculty development, can assist in overcoming resistance.

# JUSTIFYING THE PROGRAMME

*Justifying the Programme* relates to the increasing demand for public accountability. The aim of activities in this dimension is to defend the current practices of the programme in action and demonstrate that purposes are met. *Justifying the Programme* deals with the rationale behind it based on the leading purpose. Three elements can be distinguished in justifying the assessment programme. First the *Effectiveness of the Programme* deals with the question of whether the purposes of the programme can be achieved, by providing evidence of due practices. The second element, *Efficiency*, is concerned with the realities of limited resources and providing evidence of cost-effectiveness. The third element, *Acceptability*, relates to the dimension of *Stakeholders*. The focus of this element is on the broader framework of legislation and external stakeholder groups.

## EFFECTIVENESS

### Scientific Research

*Scientific research* on assessment components and activities is needed to support practices with sound evidence, which is in line with the prominence in medicine of the drive for evidence-based practice. Although this is a general principle which should guide the design of the programme as a whole, it comes into effect when one has to account for choices made in the programme.

#### F1 Before the programme of assessment is designed, evidence should to be reviewed.

This is a specification of general guideline II. This way the design can be informed by and based on scientific evidence and/or best practices. The relevant literature has to be reviewed in order to make state of the art decisions regarding the design of a programme of assessment or even elements of the programme.

#### F2 New initiatives (developments) should be accompanied by evaluation, preferably scientific research.

Scientific research which supports the activities in the programme of assessment is but one form of justifying the effectiveness. The domains or areas of research may be diverse as education and assessment are based on various scientific domains in humanities, social science, psychometrics and cognitive (neuro)sciences.

### External Review

Justification also requires *external review* of programmes of assessment. Assessment programmes are also shaped by the needs and wishes of external stakeholders.

#### F3 The programme of assessment should be reviewed periodically by a panel of experts.

Stakeholders within the organisation have two disadvantages when justifying the programme. First, they are not independent and may have conflicts of interest. Second, they may have developed blind spots over time.

#### F4 Benchmarking against similar assessment programmes (or institutes with similar purposes) should be conducted to judge the quality of the programme.

It is possible to determine to what degree the purposes of the programme are met. However, without comparison between programmes, it is impossible to judge whether this is the best possible programme given the circumstances.

## EFFICIENCY: cost-effectiveness

In every institution or organisation, resources - including those for assessment programmes - are limited. *Cost-effectiveness* is regarded as a desirable endeavour.

#### F5 In order to be able to justify the resources used for the assessment programme, all costs (in terms of resources) should be made explicit.

Decisions are made based on cost-effectiveness. In order to be able to make these decisions the required resources must be made explicit. Resources can be: time, money, materials, expertise, etc.

#### F6 A cost-benefit analysis should be made regularly in light of the purposes of the programme. In the long term, a proactive approach to search for more resource-efficient alternatives should be adopted.

If the programme of assessment can be made more efficient, resources can be freed up for other activities.

## ACCEPTABILITY: political-legal justification

As an assessment programmes does not exist in a vacuum, political and legal requirements often determine how (part of) the programme of assessment has to be (re)designed and justified.

#### F7 Open and transparent governance of the assessment programme should be in place and can be held accountable

With every design step one has to ask oneself: “Can I defend this, if it ends up in the media?”; “Can I explain and rationalise the actions taken?”

#### F8 In order to establish a defensible programme of assessment there should be one vision (on assessment) communicated to external parties.

Choices need to supported or at least accepted by all internal stakeholders. If it is not supported inside the organisation, it is hard to sell to the outside world.

#### F9 The assessment programme should take into account superseding legal frameworks.

When designing a programme of assessment it is important to know which laws apply e.g. university regulations, national law, international law. It might even be necessary to involve a legal department in the design.

#### F10 Confidentiality and security of information should be guaranteed at an appropriate level.

An issue, strongly related to guideline C12 about proportionality of actions based on combined data, is the use of information by third parties. Information should be stored with appropriate security measures and procedures should be in place to protect the information from being used inappropriately. Here, the proportionality principle should be heeded again. The more personal and sensitive the information, the more extensive the safety measures should be e.g. disclaimers about acquiring consent before the use of the combined data for purposes that are not specified is required at the outset.

**References**

1. Frank J (Ed.). **The CanMEDS 2005 physician competency framework. Better standards. Better physicians. Better care.** : Ottawa: The Royal College of Physicians and Surgeons of Canada.; 2005.

2. **Good Medical Practice** [http://www.gmc-uk.org/guidance/good_medical_practice/GMC_GMP.pdf]

3. ***ACGME*** [www.acgme.org]

4. Miller GE: **The assessment of clinical skills/competence/performance.** *Academic Medicine* 1990, **65:**S63-67.

5. Ward WC: **A comparison of free-response and multiple-choice forms of verbal aptitude tests.** *Applied Psychological Measurement* 1982, **6:**1-11.

6. Norman G, Tugwell P, Feightner J, Muzzin L, Jacoby L: **Knowledge and clinical problem-solving.** *Medical Education* 1985, **19:**344-356.

7. Norman GR: **Problem-solving skills, solving problems and problem-based learning.** *Medical Education* 1988, **22:**270 - 286.

8. Schuwirth LWT, Verheggen MM, Van der Vleuten CPM, Boshuizen HPA, Dinant GJ: **Validation of short case-based testing using a cognitive psychological methodology.** *Medical Education* 2000, **35:**348-356.

9. Schuwirth LWT, Southgate L, Page GG, Paget NS, Lescop JMJ, Lew SR, Wade WB, Baron-Maldonado M: **When enough is enough: a conceptual basis for fair and defensible practice performance assessment.** *Medical Education* 2002, **36:**925-930.

10. Biggs J: **Enhancing teaching through constructive alignment.** *Higher Education* 1996, **32:**347-364.

11. Swanson DB, Norcini JJ, Grosso LJ: **Assessment Of Clinical Competence: Written And Computer-Based Simulations.** *Assessment & Evaluation in Higher Education* 1987, **12:**220 - 246.
